# Evaluation of *p16^INK4α^* Hypermethylation from Liquid-based Pap Test Samples in Vietnamese Population

**Published:** 2017-09

**Authors:** Phuong Kim TRUONG, Thuan Duc LAO, Thuy Ai Huyen LE

**Affiliations:** 1. Faculty of Biology and Biotechnology, University of Sciences, Vietnam National University, Ho Chi Minh, Vietnam; 2. Faculty of Biotechnology, Ho Chi Minh City Open University, Ho Chi Minh, Vietnam

**Keywords:** Cervical cancer, Hypermethylation, MSP, *p16^INK4α^*, Vietnamese population

## Abstract

**Background:**

Human papillomavirus (HPV) infection has been considered as main cause of cervical cancer. Recently, aberrant DNA methylation at tumor suppressor genes (TSGs), leading to inactivation, has also been an early epigenetic event and cofactor in cervical carcinogenesis. This study was performed to evaluate an association between the hypermethylation of *p16^INK4α^* gene’s promoter and HPV exposure in non-invasive samples (liquid-based papanicolaous samples) in Vietnamese population.

**Methods:**

109 liquid-based papanicolaous test samples were archived and admitted from the Medic Medical Center and Au Lac Clinic Laboratory, Vietnam, from 2011–2014. Methylation-Specific-PCR (MSP) was performed to analyze methylation status from the liquid-based papanicolaous test samples identified whether HPV/or non-HPV, high-risk/low-risk HPV infection.

**Results:**

An upward trend was observed concerning the *p16^INK4α^* hypermethylation frequency in high-risk HPV infection, counting for 55.6%, and the low methylation frequency in low-risk and non-HPV infected samples, counting for 22.9%, 8.0%, respectively. The significant correlation between candidate *p16^INK4α^* hypermethylation and HPV exposure was observed (*P*<0.0001). Moreover, the odds ratio (OR) and relative risk (RR) were found in statistical significant value. (OR=5.76, 95%CI: 2.36 – 14.04, *P*<0.01; RR=3.11, 95%CI: 1.75–5.53, *P*<0.01).

**Conclusion:**

Presence of *p16^INK4α^* hypermethylation was the specific characteristic of high-risk HPV infected samples in Vietnamese population. The OR and RR values showed that the strong correlation between *p16^INK4α^* hypermethylation and high-risk HPV infection, in which increased the risk of cervical cancer. The combination of *p16^INK4α^* hyper-methylation and HPV detection based biomarker could be used in non-invasive samples obtained from high-risk cancer patients, offer significant practical advantages.

## Introduction

Current experimental and clinical data point out high-risk HPV (human papillomavirus) genotype infection, especially HPV 16 and/or 18, play a key role in the etiology of cervical cancer (1–7). Although infection with high-risk HPV genotypes is an accepted major risk factor for cervical tumorigenesis, other risk factors may contribute to the genetic alterations. Notably, cervical cancer is a multi-steps process with accumulation of genetic and epigenetic alterations in regulatory genes, leading to the inactivation or less expression of tumor suppressor genes (TSGs) or activation of oncogenes. Observation on the lack of expression of several TSGs due to the hypermethylation occurred on CpG islands (CGIs) promoter is known to be an early epigenetic event in driving carcinogenesis of various human cancers, including cancer of cervix (8–14).

*p16^INK4α^* TSGs (also known as *Cyclin-dependent kinase inhibitor 2A*, *CDKN2A*) is located at 9p21. Its encoded protein functions as a specific inhibitor of cyclin-dependent kinase 4 (cdk4) and cyclin-dependent kinase 6 (cdk6), that initiates D-dependent phosphorylation of the retinoblastoma tumor suppressor protein (Rb) (15–18). Hypermethylation of the *p16^INK4α^* promoter region has been detected in human cancers, including cervical cancer (10, 15–17, 19–21,).

To date, almost limited researches were carried on to provide whether or not an association between patterns of DNA hypermethylation and high-risk HPV infection, led to risk of cervical cancer. A better understanding of those principles will provide the most favorable to prognosis and diagnosis of cervical cancer.

Here, we performed an MSP (methyl-specific PCR) method to evaluate the frequency of *p16^INK4α^* promoter methylation in Vietnamese population and assess the association between *p16^INK4α^* promoter methylation and HPV 16 and/or 18 genotype infection. Notably, the Papanicolaou (Pap) samples, kind of non-invasive samples, collected from Vietnamese cervical cancer patients, enrolled in current study to develop non-invasive method for prognosis and early diagnosis of cervical cancer based on the detection of *p16^INK4α^* methylation status.

## Materials and Methods

### Ethics statement

We used anonymized routine specimen surplus to clinical requirements for assay validation, adhering to a governance framework agreed by and with a Medic Medical Center and Au Lac Clinic Laboratory ethics agreement relating to the use of specimen surplus to clinical needs.

### Sample collection

Totally, 109 Pap test samples, from 2011–2014, were archived and admitted from the Medic Medical Center and Au Lac Clinic Laboratory, Vietnam. All the samples were collected from patient’s diagnosis with HPV infection LightPoweriVA HPV genotype PCR-RDB Kit (Code: VA.A02-003E, Viet-A Corporation, Vietnam) was applied in HPV detection and genotyping. Subsequently, all the samples were divided into groups: non-HPV infection consisted of 48 samples; and positive-HPV infection, in which composed of 36 high-risk HPV (HPV genotype 16, 18 and other high-risk genotypes) infected samples and 25 low-risk HPV infected samples.

### DNA extraction, bisulfite modification

A total of genomic DNA was isolated from Pap samples by phenol/chloroform method. Cells obtained from Pap samples were lysed in lysis buffer (10 mM Tris-HCl pH = 8, 10 mM EDTA, 150 mM NaCl, 2% SDS) containing Proteinase K (0.1 mg/ml). Then, total of genomic DNA was isolated and purified by using standard phenol-chloroform and ethanol precipitation. The bisulfite conversion of 2 μg genomic DNA was performed using EpiTect Bisulfite Kits (Qiagen). The final precipitation was eluted in a volume of 20 μl and stored at −20 °C for further studies.

### Methylation-specific polymerase chain reaction

Two pairs of primer were used to amplify the regions of interest. One pair recognized a sequence in which CpG sites were methylated (unmodified by bisulfite treatment).

Other pair recognized a sequence in which CpG sites were unmethylated (modified to UpG treatment). The primer sequences and X °C for each annealing temperatures were noted in Table 1.

**Table 1:** Methylated and unmethylated of *p16^INK4α^* gene primer sequences.

**Primer name**	**Primer sequence (5′ – 3′)**	**X°C**	**P**
***p16* -M-F**	T**T**A**TT**AGAGGGTGGGG**CG**GA**TCGC**	63°C	149
***p16* -M-R**	**G**ACCC**CGAA**C**CGCGA**C**CG**T**AA**		
***p16* -U-F**	T**T**A**TT**AGAGGGTGGGG**TG**GA**TTGT**	56°C	150
***p16* -U-R**	**CA**ACCC**CAAA**C**CACAA**C**CA**T**AA**		

* Note: CpG islands were bold and underlined; X°C: primer annealing temperature. M: methylated, U: Unmethylated; F: Forward; R: Reverse; P: product size.

MSP assay was carried out in a total of 15 μl containing 3 μl bisulfite-modified template DNA, 0.75 unit iTaq DNA polymerase (Biorad), 0.5 μM each primer, 7.5 μl MyTaq^TM^ Mix (Bioline). Thermal cycling was initiated at 95 °C for 5 min, followed by 40 cycles of denaturation at 95 °C for 30 sec, annealing at the X °C for 30 sec, extension at 72 °C for 30 sec, and a final extension at 72 °C for 10 min (Note: X °C was the specific annealing temperature for each specific methylated or unmethylated primer). The PCR products were run on 2% agarose gel with visualized by ethidium bromide staining. Then, MSP products were sequenced to confirm the specificity of primers, examine the efficiency of bisulfite modification and the hypermethylation status of target gene.

### Statistical analysis

Statistical analyses were performed by Medcalc® ver. 12.7.0.0. The average frequency of methylation was calculated. The association between hypermethylation of *p16^INK4α^* and HPV infected status were examined by using Chi-square test. The differences of methylation frequencies of *p16^INK4α^* among HPV infection status were considered statistically significant at *P*≤0.05. Moreover, the association between hypermethylation of *p16^INK4α^* and risk of cervical cancer was estimated by computing OR, RR and 95% confidence intervals (CI).

## Results

### Status of the promoter hypermethylation of p16^INK4α^

The frequency of the promoter methylation of *p16^INK4α^* in 48 non-HPV infected samples; 36 high-risk HPV infected samples and 25 low-risks HPV infected samples were examined by MSP. Overall, the promoter frequencies for *p16^INK4α^* in high-risk HPV, low-risk HPV, and non-HPV infected samples were 55.6% (20 of 36 samples), 8.0% (2 of 25 samples), 22.9% (11 of 48 samples), respectively. Conversely, the promoter unmethylation frequencies were 44.4% (16 of 36 samples), 92.0% (23 of 25 samples) and 77.1% (37 of 48 samples) in high-risk HPV, low-risk HPV, and non-HPV infected samples, respectively. Methylation of *p16^INK4α^* in high-risk HPV infected samples was found to be significantly higher than other two groups (*P<*0.0001) (Table 2).

**Table 2:** The methylation profile for *p16^INK4α^* gene

**Samples**	**p16*^INK4α^* n (%)**	
	**M**	**U**
**High-risk HPV infected**	20 (55.6)	16 (44.4)
**Low-risk HPV infected**	2 (8.0)	23 (92.0)
**Non-HPV infected**	11 (22.9)	37 (77.1)
***p value***	*0.0001*	

**Fig. 1:**
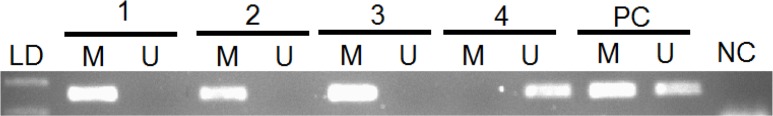
Methylated promoter of *p16^INK4α^* gene was analyzed on some clinical samples by MSP. *(The MSP product was 149/150 bp in length. (1) (2) high-risk HPV infection; (3) Low-risk HPV infection; (4) non-HPV infection; PC: positive control (standard DNA, Qiagen); NC: negative control; LD: 100 bp ladder).*

**Fig. 2:**
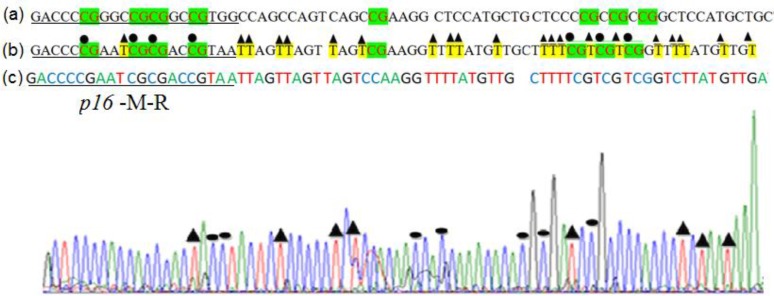
Sequencing profile of segment methylated of *p16^INK4α^*. CG sites were in the green highlight; Cytosine did not depend on the CpG sites were in yellow. (a) DNA sequence was without bisulfite modified; (b) DNA sequence was bisulfite modified; (c) The *p16^INK4α^* sequencing by using the p16-M-R primer.

The MSP products of samples hypermethylation and/or unmethylation in the promoter of *p16^INK4α^* were observed in electrophoresis with visualized by ethidium bromide staining and showed in Fig. 1. The MSP products of *p16^INK4α^* in clinical samples were observed in the band of 149 bps and 150 bps length in case of methylation and unmethylation, respectively. The sequencing of samples hypermethylated promoter region of representative sample revealed a conversion of unmethylated Cytosine, but not methylated Cytosine (Fig. 2). By sequencing, comparison between the non-bisulfite modified (Fig. 2a) and bisulfite modified (Fig. 2b), all methylated Cytosines were unchanged, marked as green characters. Otherwise, all the Unmethylated Cytosines were totally changed into Thymine in bisulfite sequence. Additionally, four methylated CpG sites were observed in methylated reverse primer, which was according to the primer designed.

### Odds ratio, relative risk for promoter hypermethylation in p16^INK4α^ gene

The odds ratio and relative risk values were computed between high-risk HPV infected group and low-risk HPV group combined with non-HPV infected group. The results show that odds ratio, relative risk values were 5.76 (95%CI=2.36 –14.04, *P*<0.01) and 3.11 (95%CI=1.75–5.53, *P*<0.01), respectively.

## Discussion

In last decades, DNA hypermethylation plays an important role in human cervical tumorigenesis. In particular, the abnormal hypermethylation in promoter region of many TSGs is associated with the inactivation of expression (23, 24). In the current study, we examined the methylation status of *p16^INK4α^* in Pap test samples by MSP method. Instead of relying on restriction enzymes that can recognize methylation sites, MSP method provide a fast approach to assess (24, 25). In hypermethylation analysis, MSP method shows an advantage in the requirement of only small quantities of DNA and sensitive to 0.1% methylated alleles of given CpG island locus and could be applied on DNA extracted from non-invasive samples (24). Noninvasive cervical screening methods such as Pap test can detect early stages of tumor development. . It contains exfoliating cells from the transformation zone of the cervix; thus, the presence of DNA in exfoliating cells has offered an opportunity to enable examination of hypermethylation analysis (7, 26–28).

Our results showed that a lower frequency of methylated *p16^INK4α^* in high-risk HPV infected samples counting for 55.6% (20 of 36 samples) when compared to a previous study, which pointed out the hypermethylation frequency of *p16^INK4α^* reached to 71.4% (55 of 77 cases) (29). Similarly, the low frequency of 17.8% (13 of 73 samples) was found in no high-risk HPV infected samples (including low-risk and/or HPV infection), which was according to another study. Additionally, we reported a strongly significant statistical association between the presence of high-risk HPV infection and hypermethylated *p16^INK4α^* (*P*<0.0001). In fact, many studies have been already correlated viral exposure to epigenetic alteration in various human cancers, including cervical cancer. High-risk HPV (such as type 16, 18) infection is necessary to cervical cancer development, only infection is not sufficient to cause of cervical cancer. Recently, growing evidence proved that epigenetic factors, such as hypermethylation, have been suggested as contributing mechanisms to cervical tumorigenesis (11, 17, 19, 20, 28, 30). Virally encoded oncoproteins, such as HPV-16 E7 associated in vitro and in vivo with the DNA methyltransferase 1 (DNMT1) considered as the enzymatic machinery involved in gene methylation. HPV-16 E7 binds to DNMT1 and precipitate, upregulate DNA methyltransferase activity (31). Thus, high-risk HPV infection could promote the silencing of *p16^INK4α^* gene, contributing to development of cervical cancer.

The hypermethylation of *p16^INK4α^* in cervical patients was significantly associated with an approximately 5.76-fold increase in the high-risk HPV infected group than compared to low-risk and/or non-HPV infected group (OR=5.76, 95%CI=2.36–14.04, *P*<0.01). This result was according to the study, which pointed out the OR=5.82 (95%CI=1.95–19.3, *P=*0.01). Concerning to RR value, high-risk HPV infection significantly increased 3.11 times in the risk of *p16^INK4α^* hypermethylation, leading to the inactivation of *p16^INK4α^* (95%CI =1.75–5.53, *P*<0.01).

Therefore, identification of HPV high-risk (such as HPV 16, 18) and hypermethylation of *p16^INK4α^* gene by contributing the risk of cervical cancer development. The MSP method for *p16^INK4α^* hypermethylation detection and HPV typing in Pap test samples could be considered as the promising candidate, non-invasive biomarkers potentially used for diagnosis and prognostic purposes in Vietnam.

## Conclusion

A higher prevalence of *p16^INK4α^* promoter hypermethylation in high-risk HPV infected samples, counting for 55.6%. On the contrary, the low frequency of *p16^INK4α^* promoter hypermethylation was found in low-risk and non-HPV infected samples. Additionally, the significant correlation between candidate gene hypermethylation and HPV exposure as well as the odds ratio and relative risk were found in the significant correlation, counting for 5.76 and 3.11, respectively were reported. The screening, which based on the combination of both high-risk HPV detection and *p16^INK4α^* gene’s promoter methylation, will be an auspicious characteristic for early prognosis and diagnosis of cervical cancer. Moreover, MSP assay done in candidate gene on the non-invasive samples (liquid-based Pap test) would provide the potential method, easily applied in clinic, to prognosis and early diagnosis of cervical cancer in Vietnamese population. In further study, the present findings require extension to a larger number of samples and series on many potential genes in order to get the profile of methylated genes related to cancer of cervix.

## Ethical considerations

Ethical issues (Including plagiarism, informed consent, misconduct, data fabrication and/or falsification, double publication and/or submission, redundancy, etc.) have been completely observed by the authors.
